# Supplementary material for: Bioactivity of Wild and Cultivated Legumes: Phytochemical Content and Antioxidant Properties
Source: Antioxidants (Basel). 2023 Apr 1;12(4):852. doi: 10.3390/antiox12040852 (PMC10135128; doi:10.3390/antiox12040852)
Supplement: Supplementary file 1 [file antioxidants-12-00852-s001.zip › antioxidants-2262345-supplementary.pdf]

SUPPLEMENTARY MATERIAL

S1: Calculated *p*-values for Tables 2, 3, 4

p-values for Table 2

Phytochemicals	L01	L02	L03	L04	L05	L06	L07	L08
TPC[1]	0.001	0.02	0.0002	0.004	0.004	0.01	0.003	0.02
TTC[2]	0.001		0.003		0.001	0.0007	0.0008	0.0008
Procyanidin B1[3]								
Chlorogenic Acid[3]	0.004		0.006					0.003
Procyanidin B2[3]			0.001					
Epicatechin[3]			0.01					
Epigallocatechin Gallate[3]			0.001					
Hesperidin[3]								
Isoquercetin[3]	0.01	0.001	0.002	0.02	0.006	0.01	0.008	0.03
Rutin[3]	0.05	0.006	0.006					0.002
Quercetin[3]	0.04		0.04	0.04	0.03			0.04
Apigenin[3]			0.03	0.05	0.03	0.007	0.05	0.05
Kaempferol[3]							0.05	

p-values for Table 3

Carotenoids	L01	L02	L03	L04	L05	L06	L07	L08
TCC Hex[1]	6.00E-06	0.00001	6.00E-06	0.000005	0.0000009	0.0000007	0.00002	0.000004
TCC DCM[1]	0.000004	3.00E-07	3.00E-07	0.000006	0.00001	0.000345901	0.0002	0.000009
Lutein[2]	0.01	0.0006		0.0003		0.00008		
β-cryptoxanthin[2]			0.00009					0.0001
α-carotene[2]	0.0001		0.0001	0.0001				0.0001
β-carotene[2]	0.005			0.003		0.05	0.02	0.0008

***p*-values for Table 4**

Sample	FRAP (mmol Fe(II)/g extract)			DPPH (mg TE/g extract)		
	Hex	DCM	MeOH	Hex	DCM	MeOH
L01	0.02	0.008	0.05		0.01	0.008
L02	0.008	0.004	0.003	0.006	0.05	0.02
L03	0.004	0.02	0.05	0.003	0.01	0.02
L04	0.002	0.003	0.0008	0.01	0.0005	0.01
L05	0.03	0.005	0.004	0.006	0.02	0.02
L06	0.03	0.001	0.004	0.001	0.04	0.02
L07	0.008	0.0006	0.000003		0.005	
L08	0.004	0.001	0.007	0.01	0.02	0.01

S2: Results of Bonferroni tests

Total Phenolic Content

Comparison of var2 by var1 (Bonferroni)

Row Mean-|

Col Mean |	L01	L02	L03	L04	L05	L06	L07
L02	0.000						
L03	0.000	0.000					
L04	0.000	0.673*	0.000				
L05	0.000	0.031	0.000	1.000*			
L06	0.000	0.001	0.000	0.175*	1.000*		
L07	1.000*	0.006	0.000	0.000	0.000	0.000	
L08	0.000	0.000	0.000	0.002	0.048	1.000*	0.000

* Insignificant (L01-L07, L02-L04, L04-L05, L04-L06, L05-L06, L06-L08)

Total Tannin Content

Comparison of var2 by var1 (Bonferroni)

Row Mean-|

Col Mean |	L01	L02	L03	L04	L05	L06	L07
L02	0.000						
L03	0.000	0.000					
L04	0.000	1.000*	0.000				
L05	0.000	0.143*	0.000	0.143*			
L06	0.000	1.000*	0.000	1.000*	0.283		
L07	0.000	1.000*	0.000	1.000*	1.000*	1.000*	
L08	0.000	1.000*	0.000	1.000*	1.000*	1.000*	1.000*

* Insignificant (L02-L04, L02-L05, L02-L06, L02-L07, L02-L08, L04-L05, L04-L06, L04-L07, L04-L08, L05-L07, L05-L08, L06-L07, L06-L08, L07-L08)

Total Carotenoid Content (Hexane)

Comparison of var2 by var1 (Bonferroni)

Row Mean-|

Col Mean |	L01	L02	L03	L04	L05	L06	L07
L02	0.000						
L03	0.000	0.000					
L04	0.000	0.000	0.000				
L05	0.000	0.000	0.000	0.000			
L06	0.000	0.000	0.000	0.000	0.000		
L07	0.000	0.000	0.000	0.000	0.000	0.000	
L08	0.000	0.000	0.000	0.000	0.000	0.000	0.000

Total Carotenoid Content (Dichloromethane)

Comparison of var3 by var1 (Bonferroni)

Row Mean-| Col Mean |	L01	L02	L03	L04	L05	L06	L07
L02	0.000						
L03	0.000	0.000					
L04	0.000	0.000	0.000				
L05	0.000	0.000	0.000	0.000			
L06	0.000	0.000	0.000	0.000	0.000		
L07	0.000	0.000	0.000	0.000	0.000	0.000	
L08	0.000	0.000	0.000	0.000	0.000	0.000	0.000

DPPH (Hex)

Comparison of var3 by var1 (Bonferroni)

Row Mean-| Col Mean |	L01	L02	L03	L04	L05	L06	L07
L02	1.000*						
L03	0.947*	1.000*					
L04	0.009	0.186*	0.250*				
L05	0.197*	1.000*	1.000*	1.000*			
L06	0.000	0.001	0.002	0.070*	0.004		
L07	1.000*	1.000*	1.000*	0.047	1.000*	0.001	
L08	0.010	0.199*	0.268*	1.000*	1.000*	0.066*	0.050*

* Insignificant (L01-L02, L01-L03, L01-L05, L01-L07, L02-L03, L02-L04, L02-L05, L02-L07, L02-L08, L03-L04, L03-L05, L03-L07, L03-L04, L03-L08, L04-L05, L04-L06, L04-L08, L05-L07, L05-L08, L07-L08)

DPPH (DCM)

Comparison of var4 by var1 (Bonferroni)

Row Mean-| Col Mean |	L01	L02	L03	L04	L05	L06	L07
L02	1.000*						
L03	0.001	0.000					
L04	0.004	0.001	1.000*				
L05	0.000	0.000	0.000	0.000			
L06	0.005	0.067*	0.000	0.000	0.000		
L07	0.049	0.004	0.065*	1.000*	0.000	0.000	
L08	0.000	0.000	0.000	0.000	0.000	0.010	0.000

* Insignificant (L01-L02, L02-L06, L03-L04, L03-L07, L04-L07)

DPPH (MeOH)

Comparison of var2 by var1 (Bonferroni)

Row Mean-| Col Mean |	L01	L02	L03	L04	L05	L06	L07
L02	0.000						
L03	0.000	0.000					
L04	0.000	0.000	0.000				
L05	0.000	0.000	0.000	0.000			
L06	0.000	0.017	0.000	0.000	0.000		
L07	0.000	0.000	0.000	0.000	0.000	0.000	
L08	0.261	0.010	0.000	0.000	0.000	0.000	0.000

FRAP (Hex)

Comparison of var3 by var1 (Bonferroni)

Row Mean-| Col Mean |	L01	L02	L03	L04	L05	L06	L07
L02	0.004						
L03	0.518*	0.124*					
L04	0.000	0.000	0.000				
L05	0.000	0.000	0.000	0.000			
L06	0.000	0.000	0.000	0.000	0.000		
L07	0.311*	0.202*	1.000*	0.000	0.000	0.000	
L08	0.000	0.000	0.000	0.000	0.080 *	0.000	0.000

* Insignificant (L01-L03, L01-L07, L02-L03, L02-L07, L03-L07, L05-L08)

FRAP (DCM)

Comparison of var4 by var1 (Bonferroni)

Row Mean-| Col Mean |	L01	L02	L03	L04	L05	L06	L07
L02	0.000						
L03	0.000	0.000					
L04	0.000	0.000	0.000				
L05	0.000	0.000	0.000	0.000			
L06	0.000	0.000	0.000	0.000	0.000		
L07	0.000	0.001	0.000	0.000	0.000	0.000	
L08	0.000	0.000	0.000	0.000	0.000	0.000	0.000

FRAP (MeOH)

Comparison of var2 by var1 (Bonferroni)

Row Mean-|

Col Mean | L01 L02 L03 L04 L05 L06 L07

---------+--

Col Mean	L01	L02	L03	L04	L05	L06	L07
L02	1.000						
L03	0.004**	0.006**					
L04	1.000	1.000	0.058				
L05	1.000	1.000	0.037**	1.000			
L06	1.000	1.000	0.013**	1.000	1.000		
L07	1.000	1.000	0.003**	0.810	1.000	1.000	
L08	1.000	1.000	0.011**	1.000	1.000	1.000	1.000

** Significant (L01-L03, L02-L03, L03-L05, L03-L06, L03-L07, L03-L08)

Antioxidants

Procyanidin B1

Comparison of var2 by var1 (Bonferroni)

Row Mean-|

Col Mean | L01 L02 L03 L04 L05 L06 L07

---------+--

Col Mean	L01	L02	L03	L04	L05	L06	L07
L02	1.000						
L03	1.000	1.000					
L04	1.000	1.000	1.000				
L05	1.000	1.000	1.000	1.000			
L06	1.000	1.000	1.000	1.000	1.000		
L07	1.000	1.000	1.000	1.000	1.000	1.000	
L08	1.000	1.000	1.000	1.000	1.000	1.000	1.000

All Insignificant

Chlorogenic Acid

Comparison of var3 by var1 (Bonferroni)

Row Mean-| Col Mean |	L01	L02	L03	L04	L05	L06	L07
L02	1.000						
L03	0.000**	0.000**					
L04	1.000	1.000	0.000**				
L05	1.000	1.000	0.000**	1.000			
L06	1.000	1.000	0.000**	1.000	1.000		
L07	1.000	1.000	0.000**	1.000	1.000	1.000	
L08	1.000	1.000	0.000**	1.000	1.000	1.000	1.000

** Significant (L01-L03, L02-L03, L03-L04, L03-L05, L03-L06, L03-L07, L03-L08)

Procyanidin B2

Comparison of var4 by var1 (Bonferroni)

Row Mean-| Col Mean |	L01	L02	L03	L04	L05	L06	L07
L02	1.000						
L03	0.000**	0.000**					
L04	1.000	1.000	0.000**				
L05	1.000	1.000	0.000**	1.000			
L06	1.000	1.000	0.000**	1.000	1.000		
L07	1.000	1.000	0.000**	1.000	1.000	1.000	
L08	1.000	1.000	0.000**	1.000	1.000	1.000	1.000

** Significant (L01-L03, L02-L03, L03-L04, L03-L05, L03-L06, L03-L07, L03-L08)

Epicatechin

Comparison of var5 by var1 (Bonferroni)

Row Mean-|

Col Mean |	L01	L02	L03	L04	L05	L06	L07
L02	1.000						
L03	0.035**	0.036**					
L04	1.000	1.000	0.035**				
L05	1.000	1.000	0.036**	1.000			
L06	1.000	1.000	0.035**	1.000	1.000		
L07	1.000	1.000	0.036**	1.000	1.000	1.000	
L08	1.000	1.000	0.036**	1.000	1.000	1.000	1.000

** Significant (L01-L03, L02-L03, L03-L04, L03-L05, L03-L06, L03-L07, L03-L08)

Epigallocatechin Gallate

Comparison of var6 by var1 (Bonferroni)

Row Mean-|

Col Mean |	L01	L02	L03	L04	L05	L06	L07
L02	0.339						
L03	0.339	1.000					
L04	1.000	1.000	1.000				
L05	0.339	1.000	1.000	1.000			
L06	0.339	1.000	1.000	1.000	1.000		
L07	0.339	1.000	1.000	1.000	1.000	1.000	
L08	0.339	1.000	1.000	1.000	1.000	1.000	1.000

All Insignificant

Rutin

Comparison of var7 by var1 (Bonferroni)

Row Mean-|

Col Mean |	L01	L02	L03	L04	L05	L06	L07
L02	0.001**						
L03	0.000**	0.000**					
L04	0.000**	1.000	0.000**				
L05	0.000**	1.000	0.000**	1.000			
L06	0.000**	1.000	0.000**	1.000	1.000		
L07	0.000**	1.000	0.000**	1.000	1.000	1.000	
L08	0.001**	1.000	0.000**	1.000	1.000	1.000	1.000

** Significant (L01-All the other samples, L02-L03, L03-L04, L03-L05, L03-L06, L03-L07, L03-L08)

Isoquercetin

Comparison of var8 by var1 (Bonferroni)

Row Mean-| Col Mean |	L01	L02	L03	L04	L05	L06	L07
L02	1.000						
L03	1.000	1.000					
L04	1.000	1.000	1.000				
L05	1.000	1.000	1.000	1.000			
L06	1.000	1.000	0.620	0.620	0.620		
L07	1.000	1.000	1.000	1.000	1.000	1.000	
L08	1.000	1.000	1.000	1.000	1.000	0.620	1.000

All Insignificant

Herperidin

Comparison of var9 by var1 (Bonferroni)

Row Mean-|

Col Mean | L01 L02 L03 L04 L05 L06 L07

---------+--

L02	0.000**						
L03	0.000**	1.000					
L04	0.000**	1.000	1.000				
L05	0.000**	1.000	1.000	1.000			
L06	0.000**	1.000	1.000	1.000	1.000		
L07	0.000**	1.000	1.000	1.000	1.000	1.000	
L08	0.000**	1.000	1.000	1.000	1.000	1.000	1.000

**Significant (L01-All the other samples)

Quercetin

Comparison of var10 by var1 (Bonferroni)

Row Mean-|

Col Mean |	L01	L02	L03	L04	L05	L06	L07
L02	1.000						
L03	1.000	1.000					
L04	1.000	1.000	1.000				
L05	1.000	1.000	1.000	1.000			
L06	1.000	1.000	1.000	1.000	1.000		
L07	1.000	1.000	1.000	1.000	1.000	1.000	
L08	1.000	1.000	1.000	1.000	1.000	1.000	1.000

All Insignificant

Apigenin

Comparison of var11 by var1 (Bonferroni)

Row Mean-|

Col Mean | L01 L02 L03 L04 L05 L06 L07

L02	1.000						
L03	1.000	1.000					
L04	1.000	1.000	1.000				
L05	1.000	1.000	1.000	1.000			
L06	1.000	1.000	1.000	1.000	1.000		
L07	0.088	0.080	0.098	0.553	0.088	0.201	
L08	1.000	1.000	1.000	1.000	1.000	1.000	0.148

All Insignificant

Lutein

Comparison of var2 by var1 (Bonferroni)

Row Mean-| Col Mean |	L01	L02	L03	L04	L05	L06	L07
L02	0.000						
L03	0.000	0.000					
L04	0.000	0.000	0.000				
L05	0.000	0.000	1.000*	0.000			
L06	0.000	0.000	0.000	0.000	0.000		
L07	0.000	0.000	1.000*	0.000	1.000*	0.000	
L08	0.000	0.000	1.000*	0.000	1.000*	0.000	1.000*

* Insignificant (L03-L05, L03-L07, L03-L08, L05-L07, L05-L08, L07-L08)

β-Cryptoxanthin

Comparison of var3 by var1 (Bonferroni)

Row Mean-|

Col Mean | L01 L02 L03 L04 L05 L06 L07

---------+--

L02	1.000						
L03	0.000**	0.000**					
L04	1.000	1.000	0.000**				
L05	1.000	1.000	0.000**	1.000			
L06	1.000	1.000	0.000**	1.000	1.000		
L07	1.000	1.000	0.000**	1.000	1.000	1.000	
L08	0.000**	0.000**	0.884	0.000**	0.000**	0.000**	0.000**

** Significant (L01-L03, L01-L08, L02-L03, L02-L08, L03-L04, L03-L05, L03-L06, L03-L07, L05-L08, L06-L08, L07-L08)

a-Carotene

Comparison of var4 by var1 (Bonferroni)

Row Mean-|

Col Mean | L01 L02 L03 L04 L05 L06 L07

---------+--

Row Mean- / Col Mean	L01	L02	L03	L04	L05	L06	L07
L02	0.000						
L03	0.003	0.000					
L04	0.678*	0.000	0.478*				
L05	0.000	1.000*	0.000	0.000			
L06	0.000	1.000*	0.000	0.000	1.000*		
L07	0.000	1.000*	0.000	0.000	1.000*	1.000*	
L08	0.005	0.000	1.000*	0.840*	0.000	0.000	0.000

* Insignificant (L01-L04, L02-L05, L02-L06, L02-L07, L03-L04, L03-L08, L04-L08, L05-L06, L05-L07, L06-L07)

β-Carotene

Comparison of var5 by var1 (Bonferroni)

Row Mean-| Col Mean |	L01	L02	L03	L04	L05	L06	L07
L02	0.000						
L03	0.000	1.000*					
L04	0.000	0.000	0.000				
L05	0.000	1.000*	1.000*	0.000			
L06	0.000	0.000	0.000	0.000	0.000		
L07	0.000	0.000	0.000	1.000*	0.000	0.000	
L08	0.001	0.000	0.000	0.000	0.000	0.000	0.000

* Insignificant (L02-L03, L02-L05, L03-L05, L04-L07)
